# Supplementary figures and images for: Through-Plane Super-Resolution With Autoencoders in Diffusion Magnetic Resonance Imaging of the Developing Human Brain
Source: Front Neurol. 2022 May 2;13:827816. doi: 10.3389/fneur.2022.827816 (PMC9109939; doi:10.3389/fneur.2022.827816)

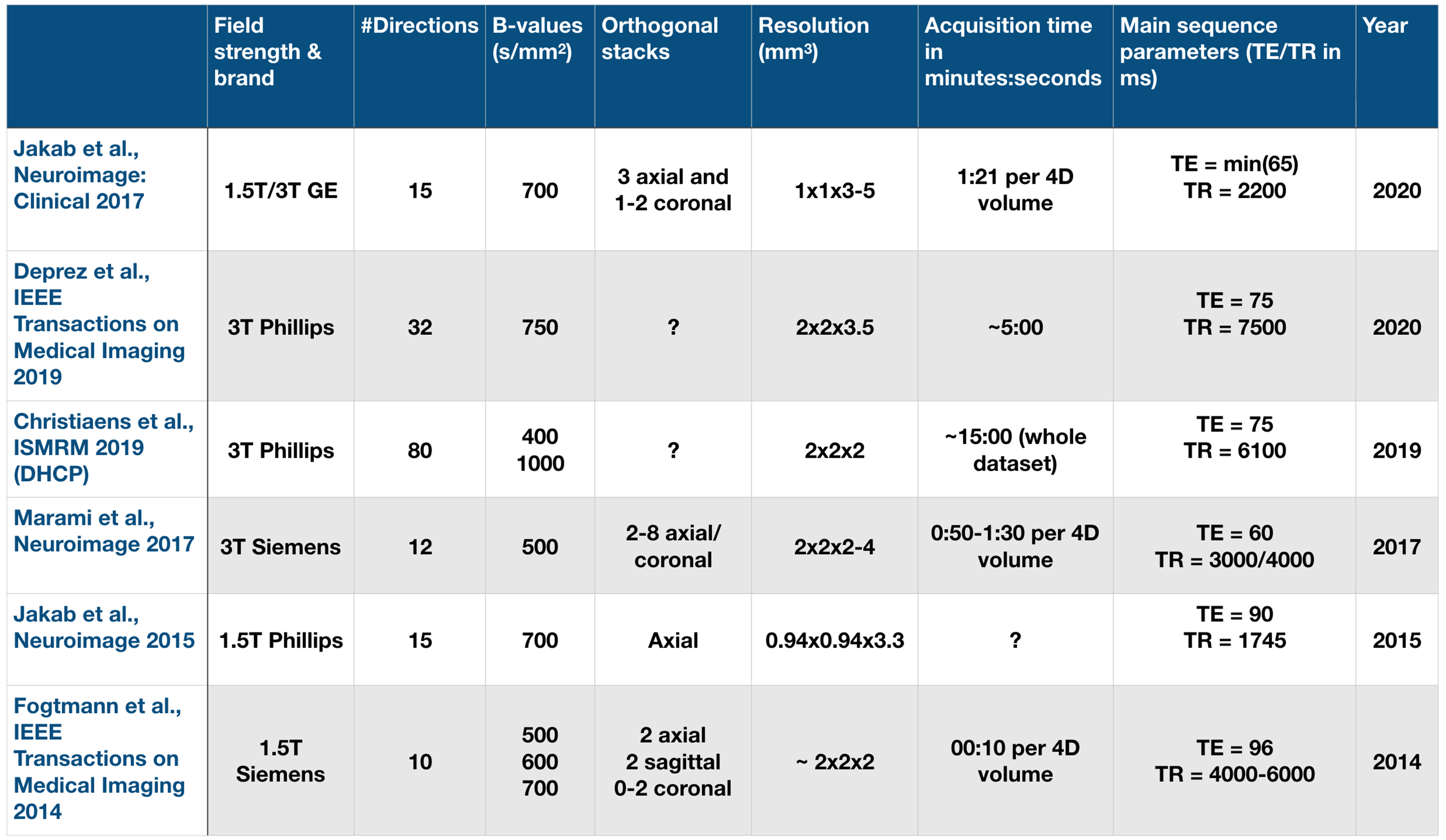

Supplement: Supplementary file 1 [file Image_1.JPEG]

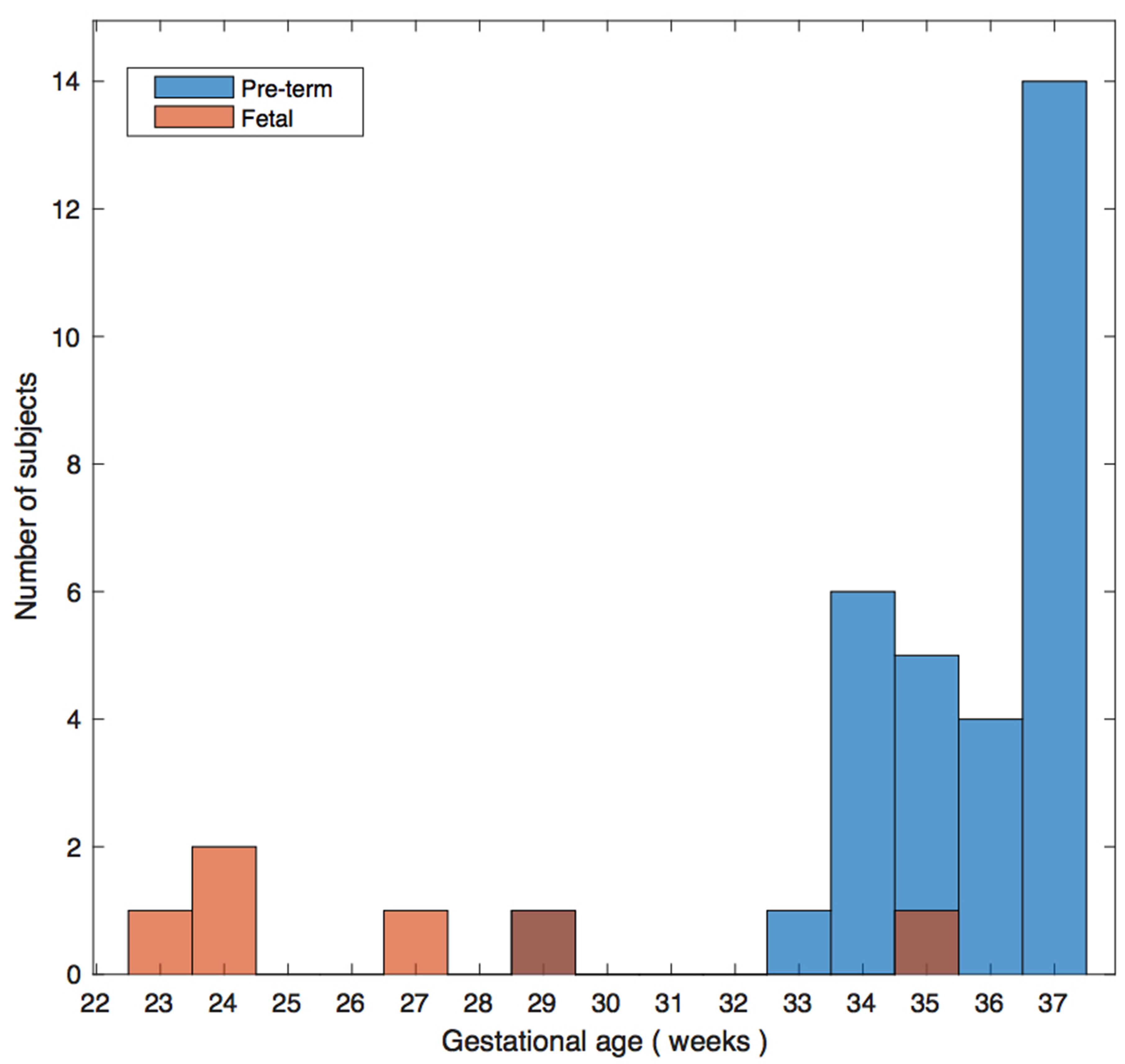

Supplement: Supplementary file 2 [file Image_2.JPEG]

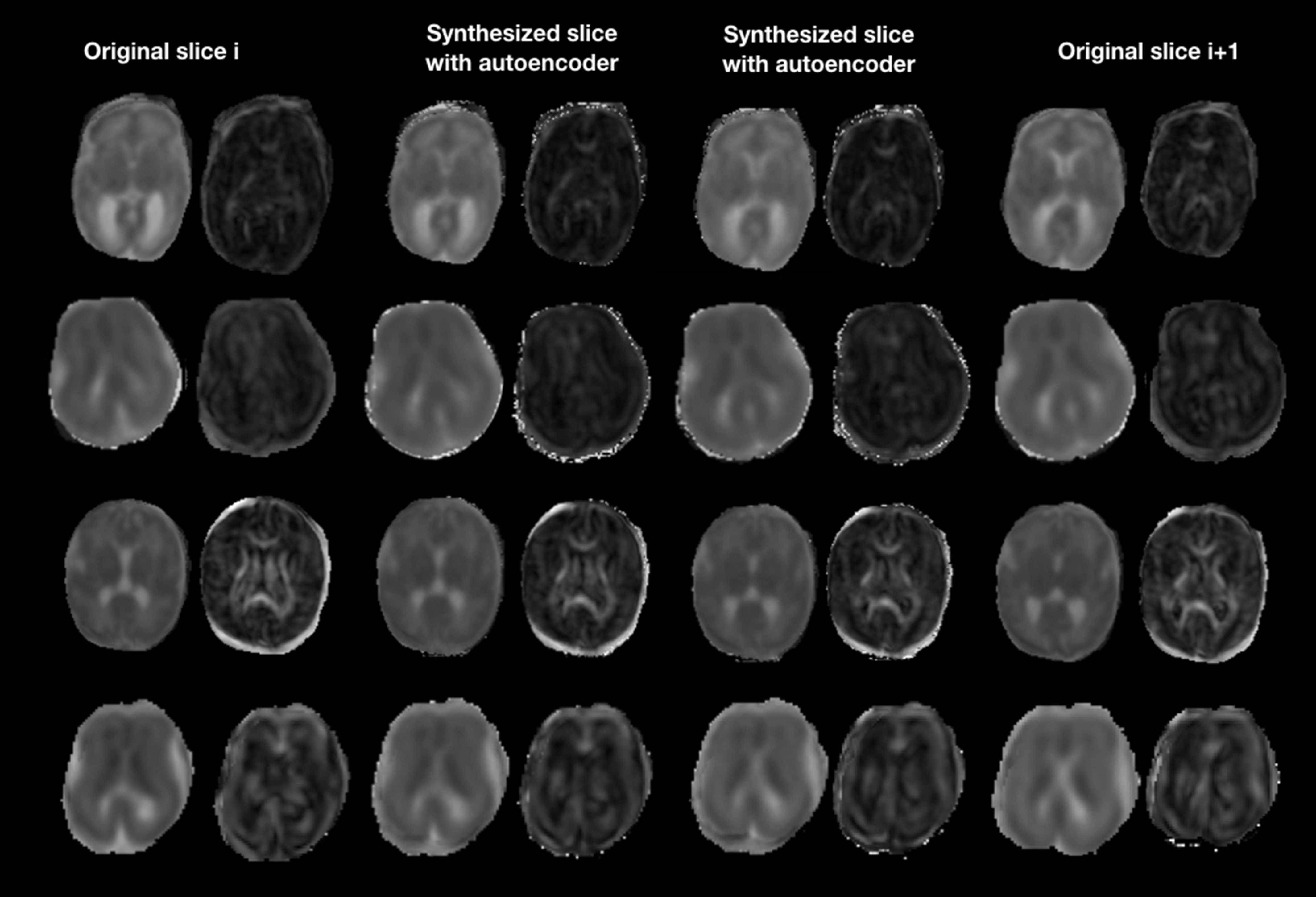

Supplement: Supplementary file 3 [file Image_3.JPEG]

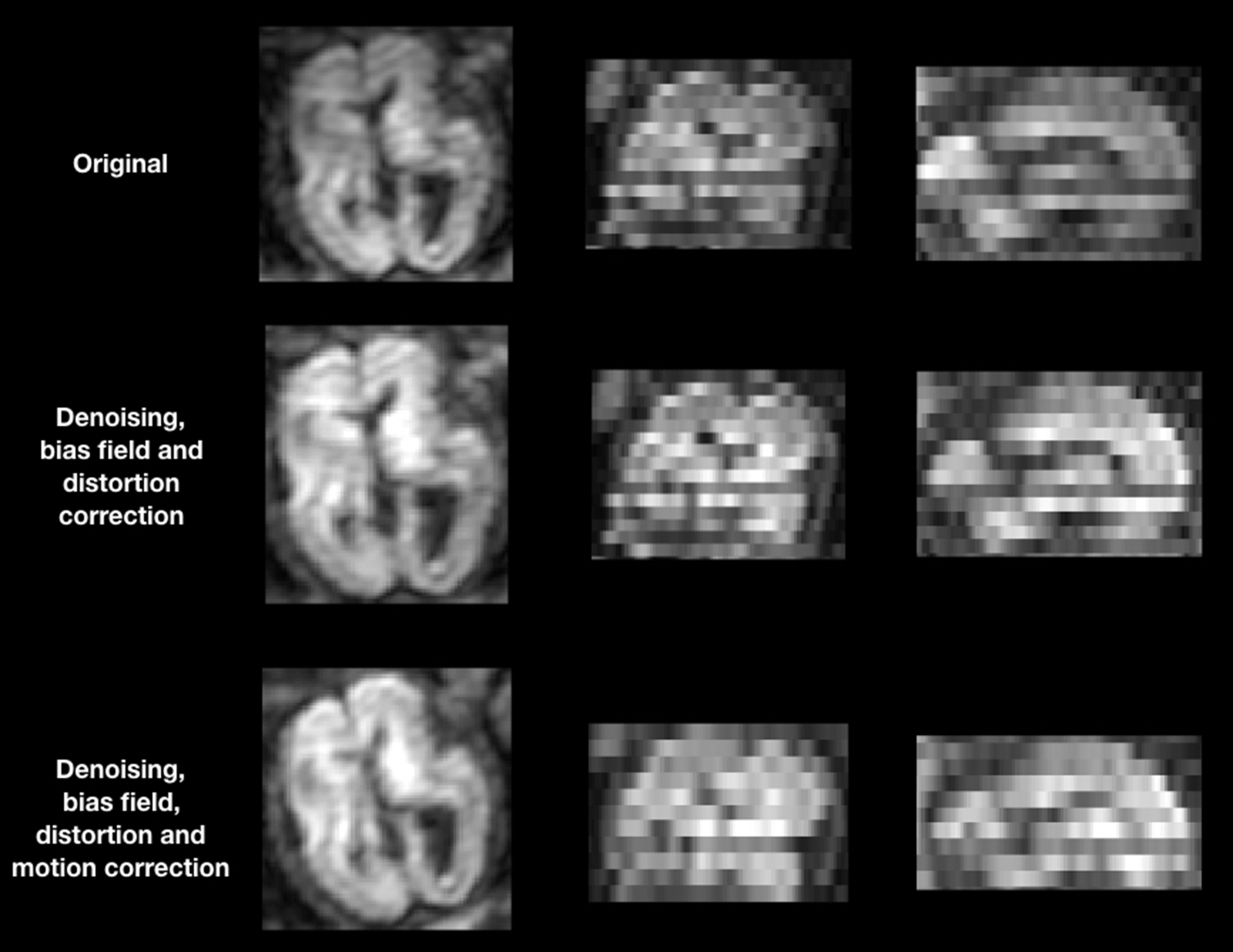

Supplement: Supplementary file 4 [file Image_4.JPEG]

GT

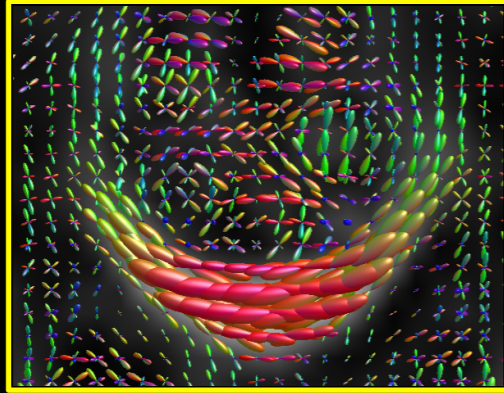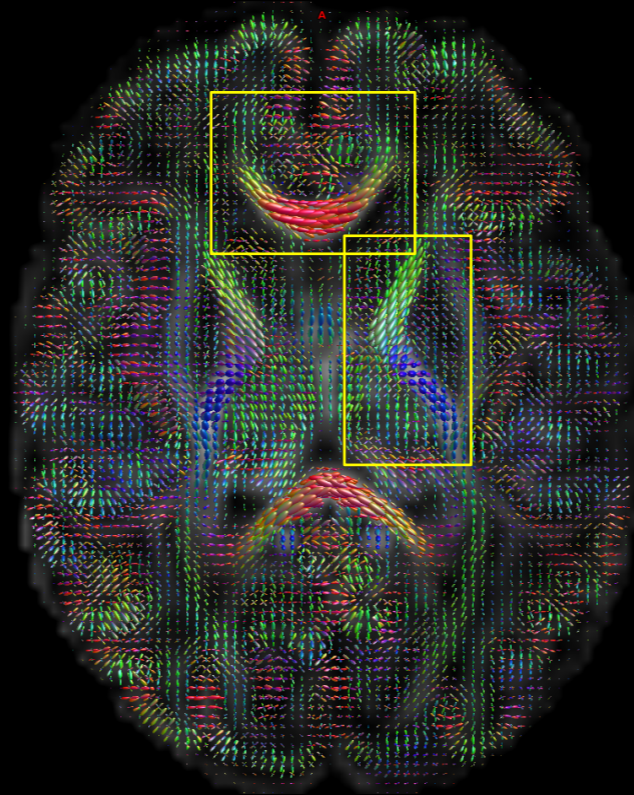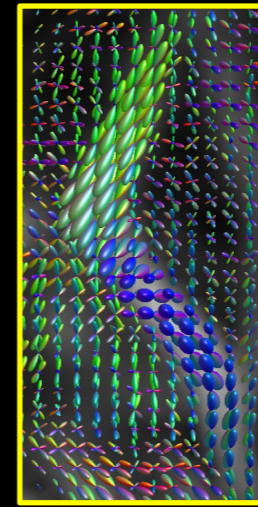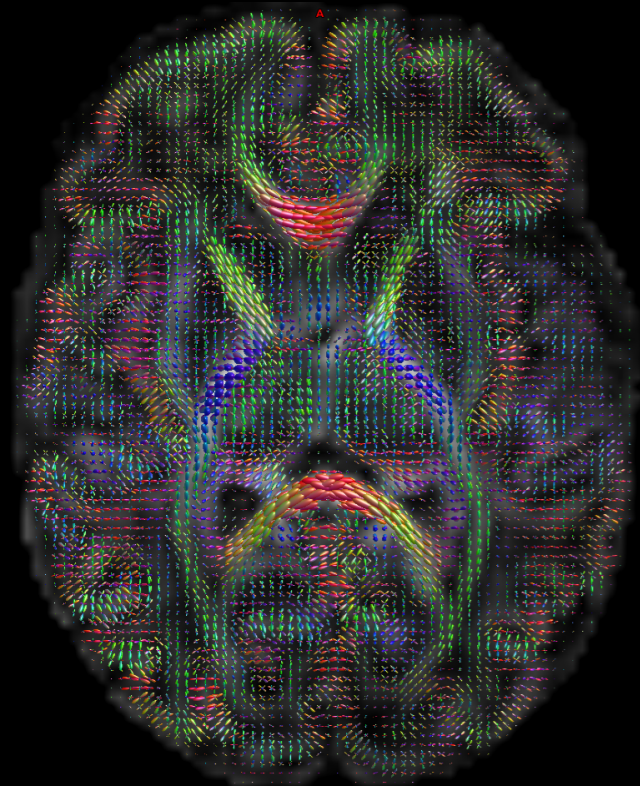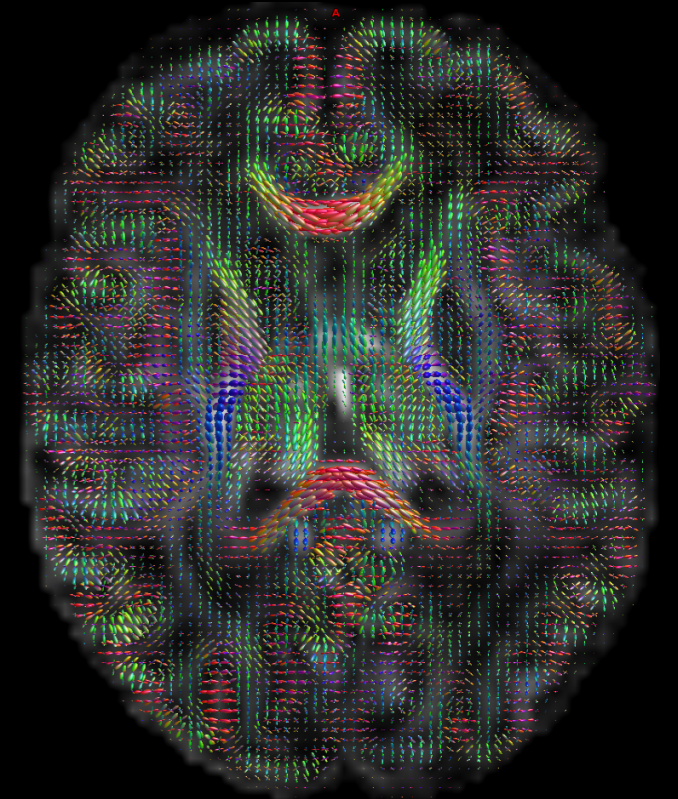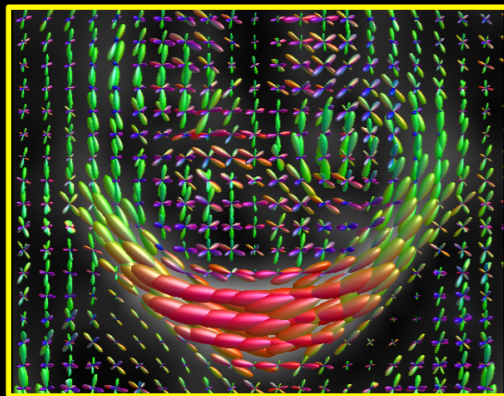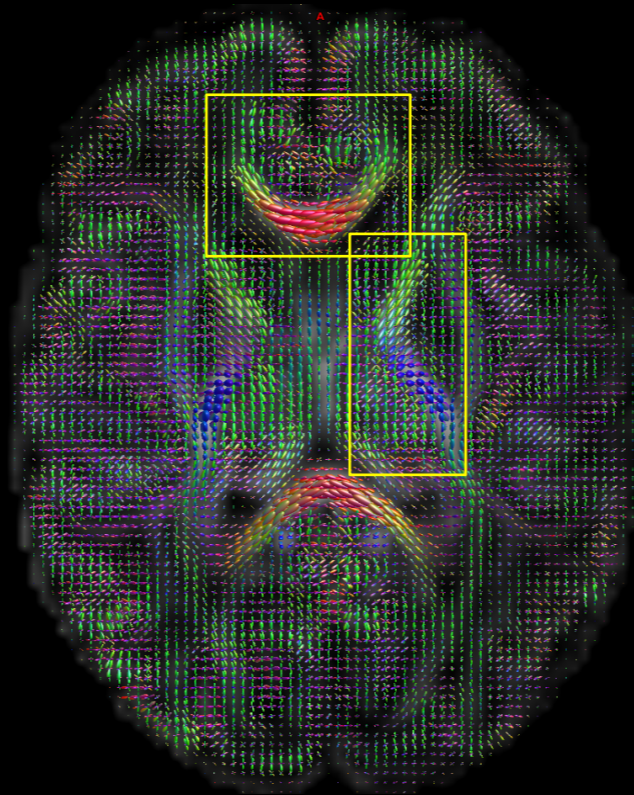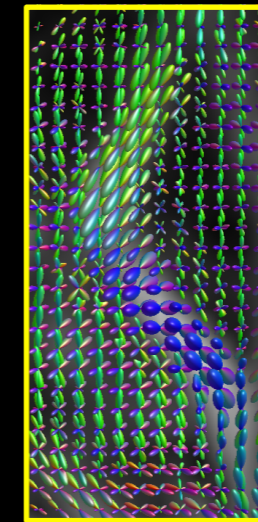

AE-1

Supplement: Supplementary file 5 [file Data_Sheet_1.PDF]

**Original-1**

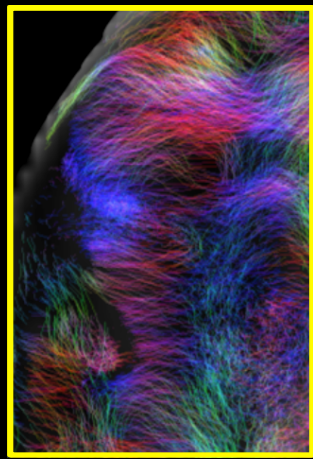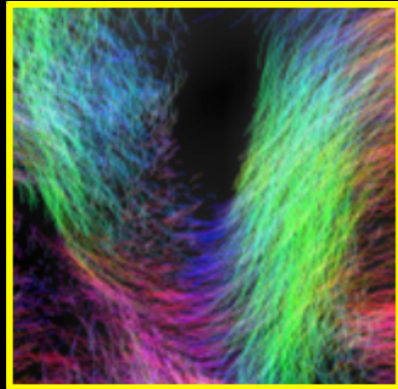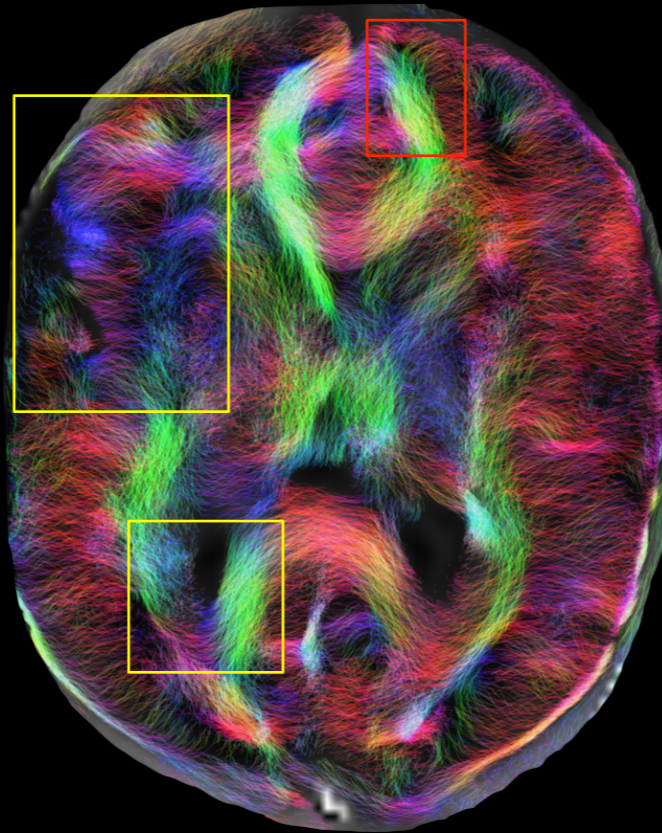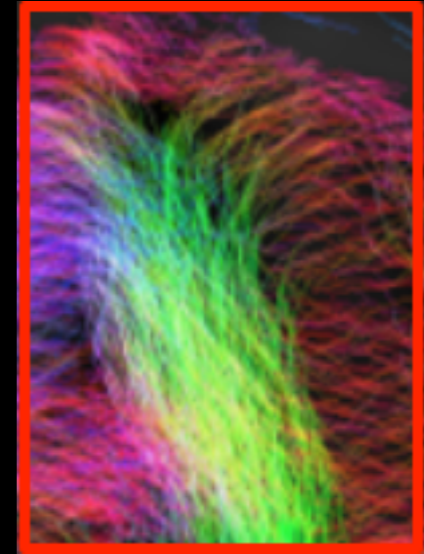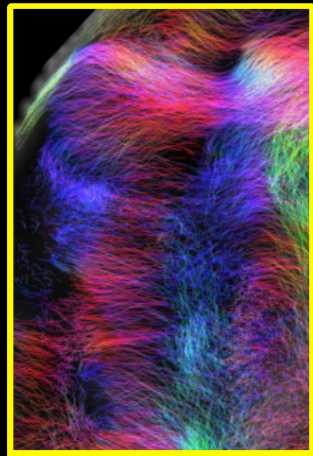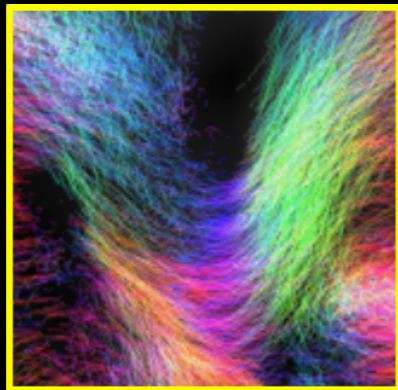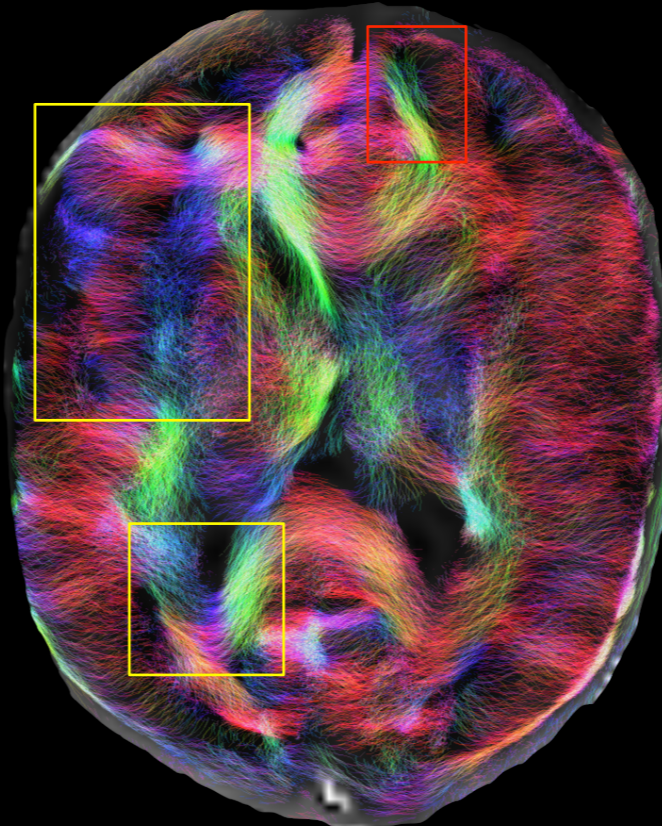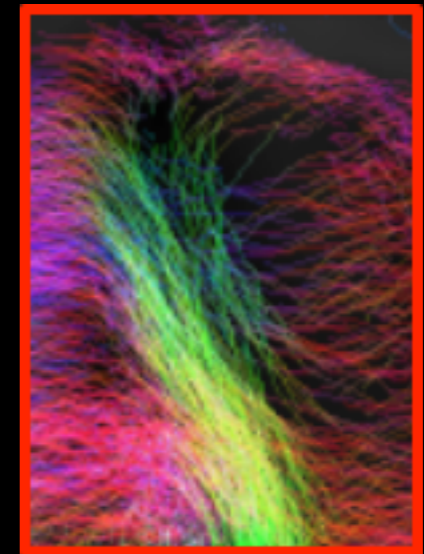

**AE-1**

Supplement: Supplementary file 6 [file Data_Sheet_2.PDF]
